# Now You See One Letter, Now You See Meaningless Symbols: Perceptual and Semantic Hypnotic Suggestions Reduce Stroop Errors Through Different Neurocognitive Mechanisms

**DOI:** 10.3389/fnins.2020.600083

**Published:** 2021-01-12

**Authors:** Rinaldo Livio Perri, Valentina Bianco, Enrico Facco, Francesco Di Russo

**Affiliations:** ^1^Department of Movement, Human and Health Sciences, University of Rome “Foro Italico,” Rome, Italy; ^2^IRCCS Fondazione Santa Lucia, Rome, Italy; ^3^Laboratory of Cognitive Neuroscience, Department of Languages and Literatures, Communication, Education and Society, University of Udine, Udine, Italy; ^4^Studium Patavinum, Department of Neurosciences, University of Padova, Padua, Italy; ^5^Inst. F. Granone—Italian Center of Clinical and Experimental Hypnosis, Turin, Italy

**Keywords:** hypnosis, hypnotizability, Stroop, EEG, ERP

## Abstract

Compelling literature has suggested the possibility of adopting hypnotic suggestions to override the Stroop interference effect. However, most of these studies mainly reported behavioral data and were conducted on highly hypnotizable individuals. Thus, the question of the neural locus of the effects and their generalizability remains open. In the present study, we used the Stroop task in a within-subject design to test the neurocognitive effects of two hypnotic suggestions: the perceptual request to focus only on the central letter of the words and the semantic request to observe meaningless symbols. Behavioral results indicated that the two types of suggestions did not alter response time (RT), but both favored more accurate performance compared to the control condition. Both types of suggestions increased sensory awareness and reduced discriminative visual attention, but the perceptual request selectively engaged more executive control of the prefrontal cortex (PFC), and the semantic request selectively suppressed the temporal cortex activity devoted to graphemic analysis of the words. The present findings demonstrated that the perceptual and the semantic hypnotic suggestions reduced Stroop errors through common and specific top-down modulations of different neurocognitive processes but left the semantic activation unaltered. Finally, as we also recruited participants with a medium level of hypnotizability, the present data might be considered potentially representative of the majority of the population.

## Introduction

The American Psychological Association (APA) defined hypnosis as “a state of consciousness involving focused attention and reduced peripheral awareness characterized by an enhanced capacity for response to suggestion” (Elkins et al., 2015). This definition emphasizes the role of attention and suggestions in hypnosis, and neurocognitive research has shown a growing interest in hypnosis and hypnotic suggestions (for reviews, see Gruzelier, 1998; Vanhaudenhuyse et al., 2014), being this topic very important to understand brain mechanisms involved in attention and motor control (for a review, see Oakley and Halligan, 2013). Among these studies, there is a promising field suggesting the possibility of reducing automatic cognitive processes through hypnosis (for a review, see Lifshitz et al., 2013). For this purpose, the Stroop effect (i.e., slow speed and low accuracy in naming the ink color of an incongruent color word) represents a “gold standard” as it is supposed to prevent learning effects, and it has been shown that posthypnotic suggestions may reduce conflict between the automatic (the reading) and intentional (naming the ink color) processes. In particular, the group of Raz and colleagues was the first to provide a compelling demonstration that hypnotic suggestions to see words as meaningless symbols can reduce (Raz et al., 2005, 2006, 2007; Raz and Campbell, 2011) or even eliminate (Raz et al., 2002, 2003) the Stroop effect in highly hypnotizable individuals (highs). However, despite some authors corroborating these findings (Parris et al., 2012), others failed to replicate them (Hung and Barnier, 2005) or even reported a deterioration in accuracy during hypnosis (Nordby et al., 1999). A few studies attempted to provide neurophysiological explanations of the effects (Nordby et al., 1999; Raz et al., 2005; Casiglia et al., 2010; Zahedi et al., 2017, 2019). In particular, an event-related potential (ERP) study by Nordby et al. (1999) reported a reduced P3a component (associated with detection of novelty) in highs during the Stroop test, but the effects were related to hypnotizability level and not to the suggestion given that they were common to baseline and hypnosis conditions. In the Stroop experiment by Casiglia et al. (2010), the posthypnotic suggestion of inability to read removed the incongruency effect on the N400 component, as also confirmed by Zahedi et al. (2019), which proposed a semantic locus of the effect and enhanced executive control associated with the increased frontal N1. Similar findings were obtained by the same authors in an electroencephalographic (EEG) frequency study reporting increased frontal theta and beta power (Zahedi et al., 2017). On the other hand, Raz et al. (2005) combined EEG and neuroimaging data to describe the neural effects of the posthypnotic suggestion to see Stroop words as nonsense strings. Authors reported decreased activity in the cuneus and the anterior cingulate cortex (ACC), pointing at hypnotic alterations at the level of visual processing in addition to conflict detection. However, the EEG–functional magnetic resonance imaging study of Egner et al. (2005) did not support such findings as they observed increased ACC activity for highs when compared to low hypnotizables (Lows) and a decrease in functional connectivity (EEG coherence) between frontal–midline and left frontal lateral sites. The results were interpreted in terms of a functional dissociation of conflict monitoring and cognitive control processes in highs.

The current debate on the neurophysiological mechanisms affected by hypnotic suggestions in the Stroop task is hindered by several methodological limitations, including the scarce number of participants or EEG electrodes and, more importantly, the recruitment of highs only, making the reliability and generalizability of the findings rather weak. As a consequence, the question of whether these results reflect only hypnotic effects among “special” selected individuals is still open. Regarding the locus of the effects, it is noteworthy that the types of hypnotic suggestions were different across studies (e.g., reading words as meaningless, inability to read, perceptual alterations). As it is now acknowledged that hypnotized participants’ brain activity mostly reflects what participants “do” during hypnosis (Kihlstrom, 2003) and that participants may produce similar responses in different ways (Woody and McConkey, 2003) as a function of the received suggestion (Lynn, 2007), there is a need to clarify if different hypnotic suggestions alter common or specific stages of stimulus processing when solving the Stroop conflict. To carefully test these hypotheses, two conditions are needed: (i) neural measures with high temporal resolution to include both prestimulus and poststimulus stages of processing and to consider activity from distributed areas of the brain and (ii) neuropsychological models of the reading processing system need to be considered.

The present study aims to target different stages of reading processes by adopting a within-subject design and providing two hypnotic suggestions that might affect sensory processing (*perceptual* suggestion) or semantic integration (*semantic* suggestion). Our hypothesis is that the Stroop behavioral performance may benefit from both hypnotic suggestions to the same extent, and this benefit should emerge through reduction in the interference and inhibition effects when compared to the no-hypnosis condition. For the underlying neurophysiological mechanisms, the two suggestions are expected to produce similar alterations in the prestimulus stage of processing, especially for prefrontal activity (indexed by the pN component; e.g., Ragazzoni et al., 2019) reflecting cognitive effort during the task preparation (for reviews, see Di Russo et al., 2017; Perri and Di Russo, 2017; Perri, 2020b). On the other hand, the two suggestions might produce specific ERP alterations in the early or late stages of poststimulus processing, with the perceptual one affecting occipital activity (the visual P1 and N1) and the semantic one producing effects starting from the frontal N300/N400 component associated with conflict detection (Liotti et al., 2000; Badzakova-Trajkov et al., 2009; Szűcs and Soltész, 2010; Sahinoglu and Dogan, 2016). In addition, in the present study, we test the involvement of the activities associated with the anterior insula (aIns) in light of recent investigations showing the contribution of this region to early prefrontal ERPs in visual tasks (Perri et al., 2014b, 2015, 2017, 2018a,b, 2019a; Ragazzoni et al., 2019). The aIns is particularly relevant for the purpose of the present study in that the activity of this region is commonly associated with sensory awareness and decision-making (for reviews, see Craig, 2002, 2010) and supports the perceptual alterations induced by hypnosis (Perri et al., 2019b). Compared to previous studies, further innovations consist in the administration of suggestions during alert hypnosis instead of posthypnotic suggestions and the recruitment of participants regardless of their responsiveness to hypnosis. In fact, investigations restricted to these individuals cannot be considered representative of the majority of subjects, making it better to include both high and medium hypnotizables (Jensen et al., 2017; Perri et al., 2020). In a previous study on the Stroop task in highs, the participants with a score of 10–12 on the French version of the Harvard Group Scale of Hypnotic Susceptibility Form A (HGSHS-A) and 9–11 on the French version of the Stanford Hypnotic Susceptibility Scale Form C were less than 6% of the population (Augustinova and Ferrand, 2012), indicating two main limitations of studies based on highs only: (a) the extremely strict selection of participants not reflecting the general population and (b) the possible variability of sample features since other studies report a rate of highs of 31% in the French version of the Harvard scale (Anlló et al., 2017) and 27.5 and 36.5% of subjects scoring 9–11 in the Italian and Mexican versions of Stanford Form C, respectively (no published French normative data are available to our knowledge) (De Pascalis et al., 2000; Sánchez-Armáss and Barabasz, 2005). Furthermore, these scales are based on a construct of suggestibility, a more and more questioned feature of hypnotizability (Kirsch et al., 2000; Weitzenhoffer, 2002; Tasso and Perez, 2008; Wagstaff et al., 2008; Facco et al., 2017; Testoni et al., 2020).

It is noteworthy that hypnotizability differences in Stroop performance were also documented in the baseline condition, with lows suffering more interference than highs and the latter performing similar to mediums (Rubichi et al., 2005). This observation further suggests the need to include mediums when drawing inferences from hypnotic experiments. Further, findings on the use of hypnosis in such cognitive tasks are important for different domains of application: in fact, the possibility to heighten the top-down control to counteract automatic intentions and behaviors would be crucial for psychotherapy. For example, automatic processes play a key role in addictions (e.g., Wiers et al., 2017), and the use of hypnosis was suggested as promising for such disorders (Katz, 1980; Lewith, 1995; Flammer and Bongartz, 2003).

## Materials and Methods

### Participants

Seventeen healthy volunteers [13 females; mean age = 23.5 (SD = 6.7) years] participated in the study. They were recruited from the student population at the University “Niccolò Cusano.” We determined the sample size for the main 2 × 2 × 3 (suggestion × condition × category) repeated-measures analysis of variance (RM-ANOVA) design of the present study with G^∗^Power software (Faul et al., 2009) using the “as in SPSS” option for estimating effect size from partial η^2^ (η^2^p). The expected effect size f(U) was set at 0.72 based on a previous study evaluating the effects of suggestion on Stroop interference (Augustinova and Ferrand, 2012). The α level was set at 0.05, and the desired power (1−β) was 90%: results indicated 15 as the minimum number of subjects to recruit in the present experiment.

Participants had no previous experience with hypnosis and no history of neurological or psychiatric disorders. The study was approved by the institutional review board of the University of Rome “Foro Italico” and was conducted in accordance with the ethical standards of the 1964 Declaration of Helsinki. All participants gave their written informed consent.

The sample’s hypnotic susceptibility score, as defined by the HGSHS-A (see below for details), was 7.1 (SD = 1.9), indicating moderate hypnotic susceptibility. Considering the canonical groupings of hypnotizability, we also identified two subgroups of highs (*n* = 6; HGSHS from 9 to 12, mean = 9.5) and mediums (*n* = 11; HGSHS from 5 to 8, mean = 5.8).

### Task and Stimuli

We adopted a classic version of the manual Stroop task where word stimuli of three distinct categories were presented 0.5 cm above a white fixation cross (diameter 0.15° × 0.15° of visual angle) in the center of a gray computer screen. Stimuli for the congruent and incongruent categories were Italian translations of the following words: BLUE, RED, YELLOW, and GREEN (BLU, ROSSO, GIALLO, VERDE). Stimuli for the Neutral category were TIME, HIT, EPOCH, and RIGID (TEMPO, COLPO, EPOCA, RIGIDO) chosen to match the length of the Italian color–word stimuli. The words subtended approximately 1° visual angle horizontally and 0.3° vertically. Participants were instructed to respond by pushing one of four buttons on the keyboard with the index and middle fingers of both hands. Each button corresponded to one of the four colors. Participants were instructed to constantly look at the fixation cross and respond as quickly and accurately as possible by pushing the colored button matching the ink color of the word stimulus. Stimuli appeared for 750 ms, and the interstimulus interval ranged from 1.5 to 2.5 s. Congruent, neutral, and incongruent trials were randomly and equally presented (0.33 probability). A total of 108 stimuli were provided in each run, and the whole task consisted of three runs for a total of 324 stimuli (108 for each category).

### Procedure

For each volunteer, participation in the experiment consisted of three sessions. In the first session, the individual level of hypnotic susceptibility was assessed, and in the second and third sessions, EEG activity was recorded while subjects performed the Stroop task.

Hypnotic susceptibility was measured using the Italian version of the HGSHS-A. The 12 suggestions from the HGSHS-A were administered orally by an author (PR) who is a licensed hypnotherapist, and participants were given a response booklet and asked to report their experience filling in the score forms. The objective score form was considered for the determination of the participants’ individual score, following the standard procedure described by Shor and Orne (1962). For each of the first 11 items, a score of one was assigned if the subject reported having experienced the suggested response; otherwise, the assigned score was zero. For the 12th item (posthypnotic amnesia), a score of 1 was assigned if fewer than four items had been reported in the response booklet before amnesia was lifted, and otherwise, a score of zero was assigned.

The second and third sessions were repeated about 1 week apart. In each session, participants were asked to perform the Stroop task in both the control (C) and the alert hypnosis (H) conditions (hereafter hypnosis). In the latter condition, the perceptual or semantic suggestion was verbally administered after a formal hypnotic induction procedure (see Perri et al., 2019b for details). The participant’s hypnotic condition was checked by observing the presence of external signals as described by Casiglia et al. (2006). The order of the two suggestions, such as the order of conditions in each session (C-H, H-C), was counterbalanced across subjects. The two suggestions were provided according to the following scripts.

#### Perceptual Suggestion (Modified From Iani et al., 2006)

“Very soon you will be in front of the computer screen, ready to play a computer game. In a moment, I will count from one to three. When I say three, you can open your eyes and remain hypnotized and ready to look at the words that will appear in the middle of the screen. Your gaze will be captured, like a magnet, by the central letter of each word. Your attention will be completely absorbed by the central letter, which will appear as very bright. Any other letter of the word will appear deformed, blurred, less luminous, and farther away from the central letter. These letters are irrelevant, and you are not interested in perceiving them. You will be able to attend to the central letter only. The letter will be printed in one of four ink colors: red, blue, green, or yellow. Your job is to quickly and accurately press the key that corresponds to the ink color shown. When I say three, you can open your eyes and play the game in a fast and automatic way. You will be active and energetic, nothing will disturb you, and you will find that you can play it easily and effortlessly while still being in hypnosis. One, two, three.”

#### Semantic Suggestion (Modified From Raz et al., 2006)

“Very soon you will be in front of the computer screen, ready to play a computer game. In a moment, I will count from one to three. When I say three, you can open your eyes and remain hypnotized and ready to look at the meaningless symbols that will appear in the middle of the screen. They will be characters of a foreign language that you do not know, and you will not attempt to attribute any meaning to them. This gibberish will be printed in one of four ink colors: red, blue, green, or yellow. You will look straight at the unknown words and crisply see all of them. Your job is to quickly and accurately press the key that corresponds to the ink color shown. When I say three, you can open your eyes and play the game in a fast and automatic way. You will be active and energetic, nothing will disturb you, and you will find that you can play it easily and effortlessly while still being in hypnosis. One, two, three.”

When the Stroop task was over, participants were asked to close their eyes and to restore their normal visual and reading skills before being completely dehypnotized. After a short interview on the subjective experience of hypnosis and a few minutes break, participants started the control Stroop or left the laboratory according to the order of conditions. During the interview, the experimenter asked the following questions to the participants: *How did you feel during hypnosis? Did something bother you? Do you think your performance has changed during hypnosis? Have you noticed any differences in your attention, perception, behavior, or anything else?*

In summary, the whole experiment consisted of four conditions: hypnosis and control for both the semantic and the perceptual sessions.

### EEG Recording

Participants were tested in a sound-attenuated, dimly lit room. They were comfortably seated in front of the computer screen and keyboard. The EEG signal was recorded using a BrainAmp amplifier connected with 32 ActiCap active electrodes (BrainProducts GmbH, Munich, Germany) mounted according to the 10-20 International System. The ground electrode was positioned on the left mastoid, and the active reference was on the FCz location. Electrode impedance was kept below 5 kΩ, and signals were digitized (rate of 250 Hz) and re-referenced offline to a common average reference. The eye movement artifacts were corrected using independent component analysis as introduced by Jung et al. (2000), and artifact rejection was performed to discard epochs contaminated by signals exceeding the amplitude threshold of ±70 μV. On average, less than 10% of the trials in each category were rejected because of the presence of artifacts, and they were equally distributed among hypnosis and control conditions. According to previous literature (Bianco et al., 2017; Quinzi et al., 2018), two different segmentations were adopted to look at the EEG signal in the prestimulus and the poststimulus stage of processing. For the prestimulus analysis, the signal was segmented in epochs of 2,000 ms (from −1,100 to 900 ms), with the first 200 ms serving as the baseline. Subsequently, the artifact-free segmented EEG was low-pass filtered (Butterworth cutoff frequency 20 Hz, slope 12 dB/octave), and all the Stroop trials were averaged for each condition (C and H in the perceptual and semantic sessions) as no differences in category were possible before the stimulus. For the poststimulus analysis, the signal was segmented in epochs of 1,200 ms (from −200 to 1,000 ms), with the first 200 ms serving as the baseline. The segmented signal was low-pass filtered (Butterworth cutoff frequency 30 Hz, slope 12 dB/octave), and the Stroop trials were averaged into congruent, incongruent, and neutral categories for each of the four task conditions.

### Behavioral and ERP Analysis

As for the behavioral data, response times (RTs) and percentage of errors (ERR) were calculated for the Stroop categories in all task conditions. The RTs were also used to calculate the main effects of the Stroop task as follows: facilitation (neutral minus congruent), interference (incongruent minus neutral), and inhibition (incongruent minus congruent).

Regarding the ERP analysis, to reduce the high number of possible comparisons across multiple scalp sites and time intervals, we selected the main components using the collapsed localizer procedure in which the considered conditions are collapsed (averaged) together (Luck and Gaspelin, 2017).

For the prestimulus activity, all conditions were collapsed, and only the intervals with global field power significantly different from zero for at least 40 ms were considered. Consequently, the interval from −400 to 0 ms was selected. To identify possible scalp electrodes to include in the analysis, a preliminary *t* test was performed comparing the average activity in the −400/0 ms time window between the hypnotic and control conditions. This analysis revealed significant differences over the lateral prefrontal F8 and F7 sites, which were selected for further analyses by adopting the hypnosis minus control differential waveforms. Activity over these sites well represents the pN component as described in a recent normative study by the present research group (Di Russo et al., 2019). Other prestimulus activities were not affected by hypnosis and were not further considered.

For the poststimulus ERPs, and after calculating the relative GFP, the following four intervals were identified: 80–130, 140–220, 250–350, and 600–900 ms (GFP significantly different from zero for at the least 20 ms). Within these epochs, the amplitude of each component was calculated as the peak amplitude at the electrode with maximal activity. Accordingly, the P1 and the pN1 were calculated in the earliest interval at P7 and Fpz sites, respectively. The N1 and the P180 were calculated in the second interval at P7 and F7 sites, respectively. The N300 was calculated in the third interval at Cz. The late positive potential (LPP) was calculated at Pz as the mean amplitude from 600 to 900 ms as it is a slow potential.

Analyses of the behavioral data were performed with a 2 × 2 × 3 ANOVA with suggestion (perceptual, semantic), condition (hypnosis, control), and category (neutral, congruent, incongruent) as factors, whereas only the suggestion and the condition effects were considered for the facilitation, interference, and inhibition effects.

For prestimulus ERPs, a 2 × 2 ANOVA was performed using suggestion and hemisphere (left, right) as factors, and poststimulus ERPs were submitted to 2 × 2 × 3 ANOVAs with suggestion, condition, and category as factors. For all ANOVAs, results were corrected for multiple comparisons using the Bonferroni test, and the effect sizes were calculated as η^2^p. According to Cohen (1988), η^2^*p* ≥ 0.01 was interpreted as a small effect, ≥0.06 as a moderate effect, and ≥0.14 as a large effect.

In addition, despite the comparison between classes of hypnotizability being out of the scope of the present work, behavioral values were also calculated for the subgroups of highs and mediums, and this analysis was carried out through independent group *t* tests for each variable. The same comparison was not possible for the ERP data because the low sample size led to low signal-to-noise ratio in the EEG traces. However, all behavioral data and significant ERP activities were correlated (Pearson *r*) with the HGSHS-A scores in order to test for any possible association with the hypnotizability level. The overall α level was fixed at 0.05.

#### Neuroelectric Source Imaging

Source localization analysis was conducted using the minimum-norm method (MNM) implemented in BrainStorm (Tadel et al., 2011) to better describe the spatiotemporal profile of the P180. The anatomical location of the active brain regions in this time window around the peak latency (140–220 ms) was identified using the Desikan–Killiany atlas (Desikan et al., 2006), and the relative source waveforms were extracted accordingly for the whole segmented epoch.

## Results

### Behavioral Data and Subjective Reports

Analysis of the RT showed a significant main effect of category (*F*_2_,_32_ = 37.17, *p* < 0.001; η^2^*p* = 0.69), and *post hoc* comparisons confirmed the known increase in RT for incongruent trials [652 ± 12 ms; 95% confidence interval (CI) = 612–692 ms] when compared to congruent (578 ± 10 ms; 95% CI = 547–608 ms; *p* < 0.001) and neutral trials (579 ± 9 ms; 95% CI = 552–606 ms; *p* < 0.001), whereas none of the other effects and interactions reached significant values (all *F* < 2.0, *p* > 0.05). Similarly, no significant effects of condition or suggestion emerged for the Stroop effects (interference, inhibition, facilitation; all *F* < 2.0, *p* > 0.05). On the other hand, ANOVAs on the ERR showed an expected significant effect of category (*F*_2_,_32_ = 7.53, *p* < 0.01, η^2^*p* = 0.32). *Post hoc* comparisons revealed more errors for the incongruent (8.0 ± 5.2%; 95% CI = 5.3–10.7%) than congruent (4.7 ± 3.4; 95% CI = 2.9–6.4%; *p* < 0.01) and neutral trials (6.4 ± 2.6; 95% CI = 5.0–7.7%; *p* < 0.05), and a significant effect of condition (*F*_1_,_16_ = 4.89, *p* < 0.05, Bonferroni-corrected *p* < 0.05; η^2^*p* = 0.23), indicating less errors during hypnosis (5.8 ± 3.2%; 95% CI = 4.2–7.5%) compared to control (6.8 ± 3.7%; 95% CI = 4.9–8.7%) conditions. Neither the main effect of suggestion nor the suggestion by condition interaction was significant, suggesting that both types of hypnotic suggestions favored a reduction in errors. Behavioral data are reported in Tables 1, 2.

**TABLE 1:** Mean response times (RTs) and percentage of errors (ERR) in the hypnosis and control conditions for the perceptual and the semantic suggestions (standard deviation is shown between parentheses).

	**Hypnosis**			**Control**		
	**Neutral**	**Congruent**	**Incongruent**	**Neutral**	**Congruent**	**Incongruent**
**Perceptual suggestion**						
RT (ms)	612 (71)	606 (74)	682 (85)	600 (68)	602 (71)	672 (91)
ERR (%)	5.7 (2.9)	3.9 (3.5)	7.0 (5.1)	6.6 (3.2)	5.0 (3.2)	8.7 (6.6)
**Semantic suggestion**						
RT (ms)	587 (65)	587 (74)	659 (93)	590 (62)	595 (66)	671 (105)
ERR (%)	6.5 (2.9)	4.6 (3.5)	7.3 (5.4)	6.6 (3.6)	5.2 (4.5)	8.8 (5.6)

**TABLE 2:** Stroop effects on the RT (ms) in the hypnosis and control conditions for the perceptual and the semantic suggestions indicated as the mean and standard deviation (between parentheses).

	**Hypnosis**			**Control**		
**Suggestion**	**Facilitation**	**Interference**	**Inhibition**	**Facilitation**	**Interference**	**Inhibition**
Perceptual (ms)	7 (10)	69 (51)	76 (49)	−1 (20)	71 (44)	70 (44)
Semantic (ms)	0 (22)	72 (50)	72 (52)	−4 (14)	81 (66)	76 (62)

*Facilitation (neutral minus congruent), interference (incongruent minus neutral), and inhibition (incongruent minus congruent) effects are considered.*

As introduced in Section “Materials and Methods,” behavioral data were additionally compared for the two classes of hypnotizability as well. Tables 3, 4 report the Stroop effects and the ERR for the subgroups of hypnotizability: all these data were compared between mediums and highs through independent *t* tests, but none of these comparisons reached statistical significance (all *p*’s > 0.05). Similarly, the correlational analyses between the behavioral data and the HGSHS-A scores did not yield any significant results (all *p*’s > 0.05). However, in order to support the novel finding that hypnotic effects were common to medium hypnotizables as well, additional control analyses were carried out excluding the highs. To this aim, the 2 × 2 × 3 ANOVA was repeated on the ERR data of the 11 subjects who scored from 5 to 8 on the HGSHS. Results showed the expected significant effect of category (*F*_2_,_20_ = 5.36, *p* = 0.01; η^2^*p* = 0.35), reflecting more errors for the incongruent trials, whereas the main effect of condition only approached the significant threshold (*F*_1_,_10_ = 4.0, *p* = 0.07; η^2^*p* = 0.28). The other main effects and interaction were not significant.

**TABLE 3:** Stroop effects (ms) in the hypnosis and control conditions for the perceptual and semantic suggestions.

	**Hypnosis**			**Control**		
	**Facilitation**	**Interference**	**Inhibition**	**Facilitation**	**Interference**	**Inhibition**
**Perceptual suggestion**						
Mediums	7 (10)	71 (48)	78 (44)	−2 (20)	64 (42)	62 (44)
Highs	6 (11)	66 (62)	72 (62)	0 (20)	84 (50)	84 (45)
**Semantic suggestion**						
Mediums	−2 (25)	66 (50)	65 (51)	−3 (13)	82 (75)	79 (72)
Highs	3 (19)	81 (53)	85 (58)	−6 (18)	78 (52)	72 (44)

*Subgroups of mediums and highs are considered (standard deviation between parentheses).*

**TABLE 4:** Mean percentage of errors (ERR) in the hypnosis and control conditions for the perceptual and semantic suggestions.

	**Hypnosis**			**Control**		
	**Neutral**	**Congruent**	**Incongruent**	**Neutral**	**Congruent**	**Incongruent**
**Perceptual suggestion**						
Mediums	6.3 (3.2)	4.5 (3.4)	7.8 (4.2)	7.1 (3)	5.4 (3.2)	9.2 (5.6)
Highs	4.7 (1.9)	2.7 (3.5)	5.5 (6.6)	5.7 (3.7)	4.4 (3.4)	7.7 (8.7)
**Semantic suggestion**						
Mediums	6.7 (2.8)	5.1 (3.9)	8 (5.4)	7.1 (4.1)	6 (5)	9.4 (4.9)
Highs	6.3 (3.3)	3.7 (2.8)	6 (5.6)	5.7 (2.7)	3.7 (3.3)	7.8 (7.2)

*Subgroups of mediums and highs are considered (standard deviation between parentheses).*

As for the subjective reports, no statistical comparisons were conducted as they were collected through unstructured interviews on the hypnosis experience.

### Electrophysiological Data

#### Prestimulus Activity

**FIGURE 1:**
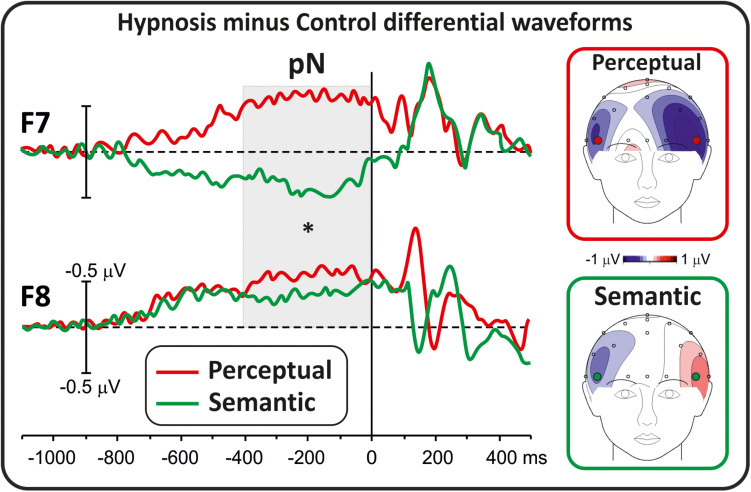
Differential ERP waveforms (hypnosis minus control) of the prestimulus activity.

Differential waveforms were obtained to test whether perceptual and semantic hypnotic suggestions affected the frontal anticipatory activity during task preparation. Figure 1 shows prestimulus differential ERPs derived from the hypnosis condition minus the control condition for the two types of suggestions. This comparison indicated that the pN component was modulated by hypnosis. In particular, the perceptual suggestion enhanced the pN over bilateral prefrontal sites (F7 and F8), whereas the semantic suggestion suppressed the pN in the left hemisphere with a slight increase over the right hemisphere. ANOVAs on the differential pN confirmed the significant effect of suggestion (*F*_1_,_16_ = 7.06, *p* < 0.05, Bonferroni-corrected *p* < 0.05; η^2^*p* = 0.30), i.e., enhanced activity for the perceptual suggestion (−0.49 ± 0.54 μV; 95% CI = −0.77 to −0.21 μV) and not semantic hypnotic suggestion (0.08 ± 0.62 μV; 95% CI = −0.41 to 0.23 μV). Effect of Hemisphere did not reach significance (*p* > 0.05).

#### Poststimulus Activity

**FIGURE 2:**
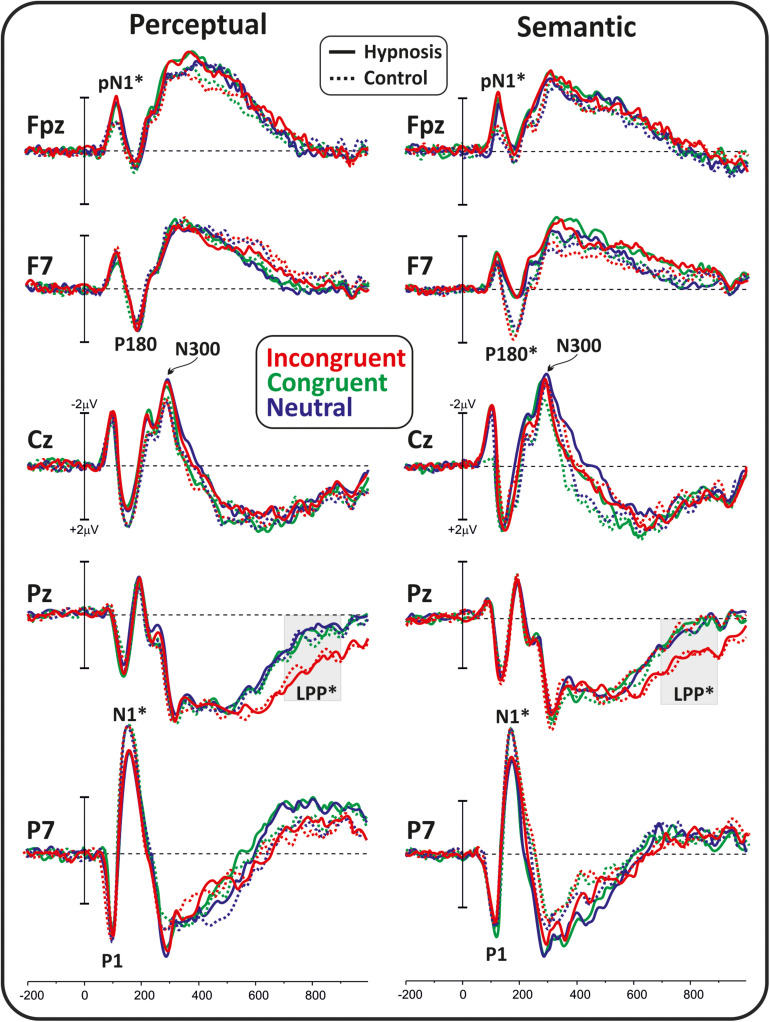
Poststimulus ERP waveforms in all conditions, suggestions, and categories. The considered components are labeled, and the significant effects are marked with stars.

**FIGURE 3:**
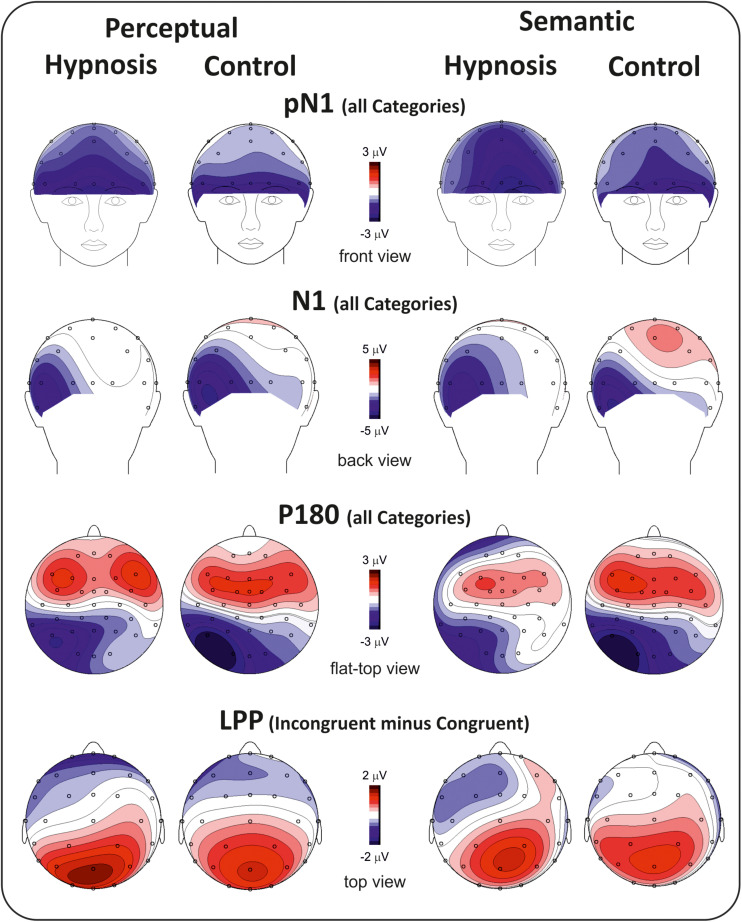
Scalp topography of the poststimulus ERP component affected by hypnosis.

Figure 2 shows the poststimulus ERPs in all conditions, suggestions, and categories, whereas Figure 3 shows the scalp topography of the significant components. The earliest component was the visual P1, peaking at 100 ms at parieto-occipital sites, which was not affected by any of the experimental manipulations (*p*’s > 0.05). At 120 ms, the visual stimuli evoked a negative peak over the frontopolar derivations corresponding to the pN1 component, which was larger during hypnosis (2.88 ± 1.24 μV; 95% CI = −3.60 to −2.16 μV) than control (2.11 ± 1.17 μV; 95% CI = −2.79 to −1.43 μV), which is regardless of the suggestion and stimuli as indicated by the significant main effect of condition (*F*_1_,_13_ = 5.2, *p* < 0.05, Bonferroni-corrected *p* < 0.05; η^2^*p* = 0.28) and the absence of significance of all other factors and interactions (all *p*’s > 0.05). The visual N1 peaked at 160 ms at parieto-occipital sites, and the ANOVA revealed a significant effect of condition (*F*_1_,_16_ = 15.7, *p* = 0.001, Bonferroni-corrected *p* < 0.01; η^2^*p* = 0.49), indicating reduced activity during hypnosis (−4.95 ± 2.05 μV; 95% CI = −6.01 to −3.89 μV) than control (−5.55 ± 2.37 μV; 95% CI = −6.76 to −4.33 μV), which is regardless of suggestion and Stroop category. None of the other effects and interactions reached significance. At 180 ms, a left-distributed positive potential emerged over the frontal lobe. This activity appeared to be suppressed by the semantic suggestion, and this observation was confirmed by the significant interaction of suggestion and condition (*F*_1_,_16_ = 5.1, *p* < 0.05; η*p*^2^ = 0.24), whereas none of the other factors and interactions reached significance. The Bonferroni *post hoc* confirmed a reduced amplitude during semantic hypnosis (1.11 ± 1.39 μV; 95% CI = 0.39–1.83 μV) that was different from its relative control (1.96 ± 1.27 μV; 95% CI = 1.30–2.61 μV; *p* < 0.5) such as from hypnosis (1.95 ± 1.35 μV; 95% CI = 1.25–2.65 μV; *p* < 0.01) and control (2.15 ± 1.62 μV; 95% CI = 1.32–2.99 μV; *p* = 0.01) of the perceptual session.

The anterior N300 and parietal LPP typically reported in the Stroop task were also observed, but none of them was affected by hypnotic conditions. In fact, the ANOVA on the N300 did not yield significant results, and only a main effect of category reached statistical significance for the LPP (*F*_2_,_32_ = 15.7, *p* < 0.001, Bonferroni-corrected *p* < 0.01; η^2^*p* = 0.49), indicating larger amplitude for the incongruent (1.83 ± 1.19 μV; 95% CI = 1.2–2.43 μV) than congruent (0.87 ± 0.85 μV; 95% CI = 0.43–1.31 μV; *p* < 0.001) and neutral stimuli (0.82 ± 0.98 μV; 95% CI = 0.31–1.33 μV; *p* < 0.001), as expected. The other main and interaction effects were not significant.

Finally, correlational analyses were conducted between the HGSHS-A scores and the amplitude of the modulated ERPs (i.e., the pN, pN1, N1, P180, LPP), but none of them reached the statistical significance (all *p*’s > 0.05).

#### Neuroelectric Source Imaging

**FIGURE 4:**
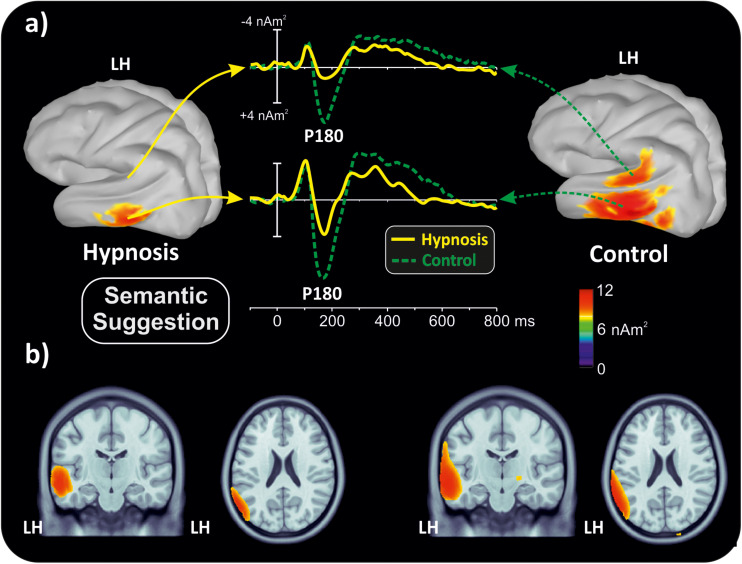
Source localization of the P180 component during semantic hypnotic suggestion and the related control condition. **(a)** Three-dimensional rendering and source time-course of the active brain regions. **(b)** P180 localization in sagittal and coronal views.

**FIGURE 5:**
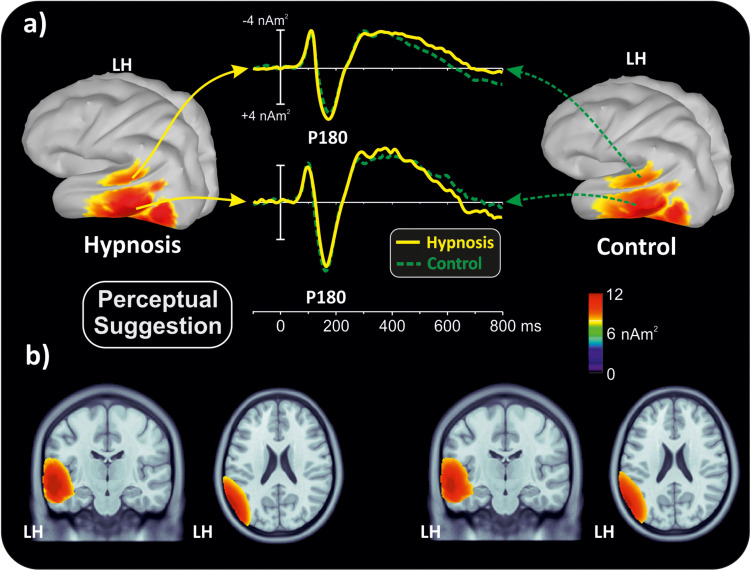
Source localization of the P180 component during perceptual hypnotic suggestion and related control condition. **(a)** Three-dimensional rendering and source time course of the active brain regions. **(b)** P180 localization in sagittal and coronal views.

With the aim of obtaining more information about the functional role and anatomical source of the P180, which is a little known component, neuroelectric source imaging analysis was carried out on the brain activity in the 140 to 220 ms interval collapsing all Stroop categories in the semantic and perceptual suggestions for comparing the hypnosis and control conditions. As shown in Figures 4, 5, the MNM approach detected two main spots of activity in the left temporal lobe: a smaller spot in the superior temporal lobe (STL) and a larger one in the middle temporal lobe (MTL). Activity at the STL spot emerged in both control conditions and with perceptual hypnosis (Figures 4, 5), whereas the MTL was recruited in all conditions to a lesser extent during semantic hypnosis. Source waveforms calculated at the center of gravity of these two areas were also extracted, showing a positive peak at 180 ms, which resembled the scalp-distributed P180. This analysis confirmed that the left temporal lobe was recruited less in semantic hypnosis, further suggesting this cortical area as the main source generating the ERP activity observed over left frontal derivations.

## Discussion

The present study aimed to clarify still open questions from the literature suggesting the intriguing possibility of suppressing automatic conflicting processes in the Stroop task using hypnosis. In particular, we addressed three unsolved topics: (1) the efficacy of different types of suggestions on Stroop performance, (2) the neurocognitive mechanisms underlying the hypnotic effects, and (3) whether the hypnosis effect was specific for highs or generalizable to mediums as well. To test these questions, we adopted a within-subject design in an ERP experiment where the perceptual and semantic hypnotic suggestions were randomly administered in a sample of individuals recruited regardless of their hypnotizability. In other words, we compared the baseline and hypnotic conditions in the same subjects instead of just describing differences between highs and lows.

Regarding the behavioral results, we observed a common advantage of the two hypnotic suggestions over control conditions reflected by reduced errors on the Stroop task. Additionally, the effect was similar across the subsamples of mediums and highs, suggesting the potential benefit of hypnosis on response accuracy existed regardless of hypnotizability level. However, different from what was expected, the hypnotic suggestions did not alter RTs or the interference effect, which was a slower response to incongruent trials than neutral trials. These data were in contrast with findings from Raz’s group (Raz et al., 2002, 2003, 2005, 2006, 2007; Raz and Campbell, 2011) but in line with studies that failed to replicate those observations (e.g., Hung and Barnier, 2005). In our opinion, the reasons might be twofold: (i) all investigations reporting reduced Stroop interference with hypnosis adopted a categorical approach consisting of comparing small groups of “special” hypnosis responders (Lows and highs). However, it was documented that the Stroop performance of these subjects differ during baseline as well (Rubichi et al., 2005), and this might be explained by the more efficient attentional focusing among the highs (Cojan et al., 2015) such as by their capacity to benefit more from hypnosis to enhance attention. In other words, the hypnosis-resistant may not be the best to compare to the hypnotic virtuosos. (ii) Unlike the previous literature, we administered suggestions in the context of alert hypnosis instead of posthypnotic suggestions, i.e., asking them to open their eyes to perform the Stroop task while remaining in hypnosis.

What emerged by unstructured interviews on the phenomenological experience of hypnosis is that for some participants it was quite difficult to quickly respond to the external stimuli while in hypnosis as they felt so good that they looked forward to closing their eyes again as soon as the Stroop was over. Perhaps, this situation might in turn help counteracting the potential benefit of enhanced attention on response speed despite the suggestion to be “active and energetic.” On the other hand, most participants reported a subjective experience of improved performance and increased attention to the visual stimuli, leading to full reception of hypnotic suggestions (e.g., “I was not able to read at all”). This situation might be interpreted as confirmation that response speed did not reflect the genuine attentional benefit of suggestions, whereas response accuracy did. According to Hung and Barnier (2005), an alternative explanation for the absence of effects on response speed along with the perceived inability to read might be the altered sense of agency, resulting in a sort of dissociation between the reading experience and the behavioral performance.

We observed both common and specific patterns of activation for the two hypnotic suggestions in the ERP data. In particular, during the expectancy stage of processing, both hypnosis conditions were associated with a slight increase in pN activity over dorsolateral prefrontal areas, with the perceptual suggestion recruiting more activity than the semantic suggestion, especially over the left hemisphere. According to the ERP literature on the pN component (see Di Russo et al., 2017 for a review) and the neuropsychological model of cognitive control developed by Cohen et al. (2004), these data might reflect an increase in executive control. This result was contrary to theories intending hypnosis in terms of loss of frontal functioning (e.g., Dietrich, 2003), but was corroborated by studies demonstrating the possibility of increasing executive control in hypnosis when required by the task and suggestion (Huber et al., 2013; Zahedi et al., 2017, 2019; Casiglia et al., 2018, 2020). For the additional effect of perceptual suggestion, it is important to note that perception of attended stimuli is a proactively prepared process in the brain (Langner et al., 2011; Perri et al., 2014a; Bianco et al., 2020). This situation was reflected by the top-down modulation of the prefrontal cortex (PFC) over the thalamic nuclei, which serves as a gating mechanism for regulating the preparatory activities of the cortex (Skinner and Yingling, 1976; Birbaumer et al., 1990). Therefore, we may hypothesize that the perceptual, suggestion-driven increase in executive control reflects the neurocognitive mechanism allowing participants to prepare themselves to be “absorbed only by the central letter, which will appear very bright.” However, in the semantic comprehension stage subsequent to visual processing, the top-down preparatory control of the PFC was less engaged in such hypnotic suggestion.

After the stimulus appeared, the two types of suggestions affected prefrontal and occipital activity in a similar way. Compared to baseline, the hypnosis conditions enhanced the anterior pN1 and reduced the visual N1 component. As the latter reflects the perceptual process of discrimination of task-relevant features within the focus of attention (Luck et al., 1990; Vogel and Luck, 2000), it might depend on the reduced engagement of attentional resources to recognize the words’ letters. In fact, despite the different strategies engaged by the two suggestions (looking only at the central letter vs. seeing meaningless symbols), both of them required not paying attention to the stimulus content. On the other hand, pN1 activity was described by ERP and neuroimaging studies as part of a complex of prefrontal ERPs reflecting the contribution of the aIns in perceptual processing (see, e.g., Di Russo et al., 2019). In particular, the pN1 was identified as the earliest step in aIns processing associated with sensory awareness (Perri et al., 2018a, 2019a), which is the ability to report the presence of a stimulus (Rees, 2007). It is not a case that the aIns participates in the entry of the stimulus into awareness (Downar et al., 2000; Craig, 2010) and that its contribution is crucial for hypnotic modulation of consciousness and perception (Hofbauer et al., 2001; Derbyshire et al., 2004; Vanhaudenhuyse et al., 2009, 2014; Jiang et al., 2017; Perri et al., 2019b). Furthermore, the hypnosis-related pN1 increment over anterior areas supports the results of Zahedi et al. (2019), who reported a large anterior N1 for the hypnotic condition. Taken together, the results on N1 and pN1 activity suggest that hypnosis favored task absorption with a greater awareness of the stimuli, accompanied by a reduced interest in discriminating the content of the words, which is in line with the instructions provided in the two types of suggestions.

As for the specific effect of the semantic suggestion, it emerged through a huge reduction in P180 activity over the left frontal cortex. Distribution, amplitude, and latency of this component were similar across all experimental conditions except for the semantic suggestion, where the P180 activity was close to 0 μV. To the best of our knowledge, Szűcs and Soltész (2010) were the only ones to report this component in a Stroop task. They described it in terms of semantic processing, a fact in line with evidence that the semantic interpretation of words happens at approximately 160 ms (Hauk et al., 2006). Nevertheless, our neuroelectric source analysis on active brain regions approximately 180 ms suggests that the STL and MTL of the left hemisphere may be the neural source of this positive potential. This finding is in accordance with the time course of the source waveforms showing a 180-ms deactivation of the temporal cortex during semantic hypnosis (Figure 4). In other words, source analysis helped distinguish the frontal scalp distribution of the P180 from the anatomical and functional properties of this component. In fact, for the auditory ERPs originating from the temporal lobe and detected over the anterior EEG locations (Tarkka et al., 1995; Opitz et al., 1999; Albrecht et al., 2000; Ponton et al., 2002; Liebenthal et al., 2003), present data suggest that the left P180 might reflect the positive polarity of neuroelectric dipoles located in the left temporal cortex. This interpretation would be further corroborated by the tangential scalp distribution of this component (see topographic maps of Figure 3). As the MTL and STL of the left hemisphere are involved in reading comprehension (Lindenberg and Scheef, 2007; Buchweitz et al., 2009), our data on the P180 may partly support the studies associating it to interpretation of words (Hauk et al., 2006; Szűcs and Soltész, 2010). More specifically, we suggest that the P180 may participate in the neural mechanism of visual word recognition known as the “visual word form system” (Warrington and Shallice, 1980; Cohen et al., 2008), especially as a marker of the temporal activation devoted to the processing of letter strings (Cohen et al., 2000). Moreover, as the P180 is 30 ms later than visual discrimination (i.e., the posterior N1) but much earlier than the stimulus conflict detection by the ACC (i.e., the N300 component), we suggest that this component reflects a still presemantic stage of reading. If that is the case, the left temporal activity at 180 ms may reflect the engagement of word-specific visual system recruitment that is needed for graphemic analysis of the words, but does not correspond to semantic activation yet as it is only a prerequisite for word comprehension. This finding is also in line with the pure alexia yielded by the disruption of the visual word form system (Cohen et al., 2008). It is worth noting that this condition may be akin to the hypnotic semantic suggestion used in this study (i.e., “you will see meaningless symbols”).

Finally, the hypnotic suggestions neither affected the anterior N300, reflecting conflict detection (e.g., Liotti et al., 2000), nor the posterior LPP enhanced by incongruent stimuli (e.g., Herring et al., 2011). The absence of hypnotic effects on these congruency-sensitive ERPs might be interpreted as a further confirmation that both types of suggestions left the word comprehension unaltered. These findings are in accordance with Augustinova and Ferrand (2012), who concluded that the hypnotic suggestions do not deautomatize word reading and do not eliminate semantic activation. In fact, reading is a highly automated and overlearned response; it probably cannot be totally suppressed. On the other hand, this conclusion contradicts the claims of Raz and colleagues that hypnosis may suppress reading (e.g., Raz et al., 2002). There may be at least two possible explanations for these discordant findings. First, there is a relevant bias of replicability favored by the limitations of previous studies of selected highs. Second, there was only use of behavioral measures and limited neurophysiological investigations, which did not allow appropriate inferences about the underlying neurocognitive mechanisms. Therefore, they were mostly hypothesized or speculated, which might explain why the present and other works failed to replicate the results of Raz’s group or to support their interpretations (Nordby et al., 1999; Hung and Barnier, 2005; Augustinova and Ferrand, 2012).

## Conclusion

To the best of our knowledge, this study is the first study that combined behavioral and neurophysiological measures to describe the contribution of different hypnotic suggestions on specific stages of decision-making to reduce conflict in a cognitive task.

Our results show that perceptual and semantic suggestions improve performance on the Stroop task. For brain activity, the perceptual suggestion to focus only on the central letter recruited more executive control of the PFC during the preparation stage, whereas the semantic suggestion to observe letters as meaningless symbols affected the graphemic analysis of the words by deactivating the left temporal cortex. Both perceptual and semantic suggestions favored an increase in sensory awareness from the aIns, together with a reduction in discriminative attention from the occipital cortex. Taken together, these findings suggest that hypnotic suggestions acted through common and specific top-down modulations of perceptual and cognitive processes. In other words, hypnosis did not suppress reading but facilitated or inhibited specific presemantic stages of stimulus processing, which in turn allowed more accurate performance. This evidence suggests the need to consider the effect of specific suggestions in further studies, rather than focus on the “hypnotic state” in general. The different brain mechanisms associated to the perceptual and semantic suggestions also allow excluding that behavioral effects were due to a generic attentional benefit of the hypnotic condition. This conclusion is also corroborated by a study demonstrating that hypnosis alone did not produce any modifications in the Stroop performance, whereas hypnosis with semantic suggestion did (Zahedi et al., 2017). It is also noteworthy that recent investigations revealed that hypnotized participants’ brain activity may undergo opposite patterns as a result of different hypnotic instructions. For example, the suggestion to ignore the external stimuli in a passive task decreased executive control by the PFC (Perri et al., 2020), whereas this control increased with the suggestion to process the external stimuli in an active task (as in this study; see also Huber et al., 2013; Zahedi et al., 2017, 2019).

It is noteworthy that the present findings might be considered potentially representative of the general population given that we included both highs and mediums, the latter having been neglected in most studies. In fact, the highs vs. lows comparison does not allow checking whether differences depend on the highs being different from the normative (Mediums) and/or the lows being extraordinarily refractory to hypnotic instructions (Kirsch and Lynn, 1995; Jensen et al., 2017; Perri, 2020a). However, because of the absence of lows and the scarce presence of highs in the present study, we cannot exclude that more careful investigations may detect the role of hypnotizability as a mediator of the suggestions effect on the Stroop performance. For the same reasons, the conclusion that hypnotic effects are common to mediums and highs needs to be corroborated by studies with larger samples, allowing more careful comparisons of behavioral and neurophysiological data.

Finally, present findings challenge the hypofrontality theories of hypnosis (e.g., Dietrich, 2003), suggesting instead that it might reflect a paradoxical metacognitive phenomenon consisting of the adaptive modulation of executive control accompanied by a reduction in the sense of agency (e.g., Dienes and Perner, 2007), in line with the APA’s definition of hypnosis that underscores the “enhanced capacity for response to suggestion.” In other words, hypnosis may be the result of an intentional management of higher-order processes and deliberate decoupling of executive control. A limitation of the present study is the absence of low hypnotizable subjects and the adoption of unstructured interviews of the phenomenological experience. In fact, low scorers would allow to understand if the present suggestions are at least in part effective for subjects with scarce responsiveness to hypnosis and to clarify the possible interaction between hypnotizability and the studied conditions. For similar reasons, careful and structured investigation of the subjective experience could help clarify if lows, mediums, and highs accept the hypnotic suggestions in different ways and whether this is related to the neurophysiological measures. Future studies recruiting larger samples could clarify these points.

## Data Availability Statement

The raw data supporting the conclusions of this article will be made available by the authors, without undue reservation.

## Ethics Statement

The studies involving human participants were reviewed and approved by the institutional review board of the University of Rome “Foro Italico.” The patients/participants provided their written informed consent to participate in this study.

## Author Contributions

RP and FD designed the study. RP, EF, VB, and FD interpreted the data and wrote the manuscript. All authors approved the submitted manuscript.

## Conflict of Interest

The authors declare that the research was conducted in the absence of any commercial or financial relationships that could be construed as a potential conflict of interest.
